# Laryngo-Tracheo-Bronchial Foreign Bodies in Children: Clinical Presentations and Complications

**Published:** 2017-05

**Authors:** Hazem-Saeed Amer, Mohammad-Waheed El-Anwar, Ashraf Raafat, Mohamed AlShawadfy, Ehab Sobhy, Samir-Attia Ahmed, Ahmed-MA Maaty

**Affiliations:** 1 *Departmen of Otorhinolaryngology, Head and Neck Surgery, Faculty of Medicine, Zagazig University, Zagazig, Egypt.*; 2 *Consultant of Otorhinolaryngology, Benha Teaching Hospital, Benha, Egypt.*; 3 *Departmen of Cardiothoracic Surgery, Faculty of Medicine, Zagazig University, Zagazig, Egypt. *; 4 *Consultant of Otorhinolaryngology, Benha Children Hospital, Benha, Egypt.*; 5 *Lecturer of Anesthesia and ICU, Faculty of Medicine, Zagazig University, Zagazig, Egypt.*

**Keywords:** Bronchoscopy, Foreign bodies, Inhalation

## Abstract

**Introduction:**

Foreign-body (FB) aspiration in the airway of children is a life-threatening clinical situation responsible for many deaths each year**. **The aim of this study was to evaluate the different clinical presentations, methods of diagnosis, types and complications of FB inhalation in the pediatric age group.

**Materials and Methods:**

The study included patients who presented with a definitive or suspicious history of FB aspiration. Detailed data for each patient were recorded concerning the age, sex, nature and site of the FB, presenting symptoms and signs, and radiological findings.

**Results:**

Fifty-six patients were enrolled in this study. The age of patients ranged from 6 months to 14 years, with a mean age of 4.5 years. Sixty percent of patients were under 3 years of age. The time interval between aspiration of foreign body and onset of diagnosis ranged from 2 hours to 5 months. Thirty-four (60.7%) patients had normal chest X-ray findings, while opaque FB was seen in eight patients (14.3%). Signs of bronchitis were seen in five patients (9%), while pneumonia and atelectasis were seen in six (10.7%) and three cases (5.3%), respectively.

**Conclusion:**

FB aspiration is a life-threatening clinical situation, with children <3 years of age being most commonly affected. FB aspiration must be considered a matter of emergency, especially in the case of organic FBs. This study aimed to increase the awareness of laryngo-tracheo-bronchial FBs, as early diagnosis and management decrease the incidence of complications and make removal of aspirated FB easier.

## Introduction

Among children, foreign-body (FB) ingestion, inhalation and aspiration represent a serious and common situation (1). Pediatric FB aspiration is a life-threatening clinical problem responsible for many deaths annually, particularly in children aged less than 2 years (2). A wide range of FBs has been reported in literature. The most frequently detected FBs are toys, sweets, batteries, jewels, rocks and magnets (3).

The clinical course and outcome of inhaled FB largely depends on the nature/type of the FB, the arrest or impaction site along the tracheobronchial tree, and potentially the availability of skilled assistance, especially in developing countries. FBs can become impacted at any site from the inlet of the larynx to the terminal bronchioles; but FBs most often become lodged in the right main bronchus in line with the trachea, thereby creating a relatively straight pathway from the larynx to the bronchus (4).

Aspiration of FB often presents with an initial history of a choking episode and coughing followed by respiratory symptoms which may cause either acute airway obstruction and death or chronic sequelae such as repeated pulmonary infections, atelectasis, and bronchiectasis (5).

Although proper history and examination are important to determine children who need investigation and removal of FBs (6), the currently used diagnostic criteria are not specific to FB aspiration and may be seen in conditions similar to FB aspiration or could be absent in some cases.

This study aimed to describe the different clinical presentations, methods of diagnosis, types and complications of FB inhalation in the pediatric age group.

## Materials and Methods

*Patients and methods:*

This prospective study was conducted at the Emergency Unit and Department of Otorhinolaryngology Head and Neck Surgery, Zagazig University Hospitals over the period from November 2013 to September 2015. Informed consent was obtained from all study subjects or their parents.

The study included 56 patients who presented with a definitive history of FB aspiration or recent onset of cough, or difficult breathing with suspicious of FB aspiration. Detailed data for each patient were recorded concerning the sex, age, type and site of the FB, presenting symptoms and signs, and interval between FB inhalation or symptom onset to hospital admission. A thorough clinical examination was performed in each patient, including a general examination and head, neck, and chest examination.

A chest X-ray was performed in all patients, but a computed tomography (CT) scan was recorded only when indicated. Routine laboratory investigations were also performed.

Rigid bronchoscopy was performed under general anesthesia for therapeutic purposes in cases in which FB was detected preoperatively and by X-ray or flexible bronchoscopy for diagnostic and therapeutic purpose in cases in which FB was not preoperatively detected. The size of the bronchoscope (external diameter) varied from 3.5 mm to 5 mm, and was selected according to the age of the patient. Bronchoscopy was performed by an otolaryngologist in all cases. In many cases the decision was made jointly by the cardiothoracic and otorhinolaryngology surgeons.

*Anesthetic management:*

1) Preoperatively: detailed data of the site and type of the FB and the time since aspiration and last medication were discussed between the surgeon and anesthesiologist; 2) Anesthetic considerations: Before induction, a detailed operative and anesthetic plan was also discussed. Inhalational induction with sevoflurane was performed in all cases after insertion of an intravenous cannula. All cases were monitored with a pulse oximeter and pericardial stethoscope and 0.02 mg/kg atropine was given. Rocuronium (0.8mg/kg) was needed in some cases to facilitate insertion of the bronchoscope. After the rigid bronchoscope was inserted via the glottis opening, the anesthetic circuit was connected to the side port of the bronchoscope to permit ventilation. Anesthesia was maintained with sevoflurane and fentanyl 1–2 µg/kg. After FB extraction and removal of the bronchoscope, the choice of ventilation during emergence was dependent on the degree of airway edema. For non-complicated cases, spontaneous ventilation that was assisted by mask ventilation as needed was adequate; 3) Postoperative considerations: uncomplicated cases were discharged early, but cases with delayed pulmonary recovery and affected pulmonary gas exchange were managed postoperatively in the intensive care unit, maintaining postoperative oxygenation. Operative data for each patient, including the site and nature of FB and also complications caused by inhalation, were recorded for each patient.

## Results

Fifty-six patients were enrolled in current study, of whom 37 were male (66%) and 19 were female (34%). The age of patients ranged from 6 months to 14 years (mean, 4.5 years). Sixty percent of patients were under 3 years of age.

The time interval between FB aspiration and diagnosis ranged from 2 hours to 5 months. Forty-two (75%) patients presented within the first week of FB aspiration, and 25 (59.5%) within the first 24 hours. Eight (14.3%) patients presented within the first month and six (10.7%) patients presented later.

All patients presenting in the first week after FB inhalation had shortness of breath only, except for one patient who presented with stridor and mild cyanosis and who required urgent management. In patients presenting later, variable degrees of productive cough, fever, and chest rhonchi were the most common signs detected on examination. This was proven radiologically to be due to bronchitis or pneumonia.

With respect to X-ray findings, 34 (60.7%) patients had normal chest X-rays – possibly because most FBs were radiolucent. An opaque FB was seen in only eight patients (14.3%). Signs of bronchitis were seen in five patients (9%), while pneumonia and atelectasis were seen in six (10.7) and three cases (5.3%), respectively.

Anesthesia and recovery was eventless in all our cases. During bronchoscopy, 57 FBs were detected in patients included in this study, as two FBs (in the form of orange seeds) were extracted from one patient; one of which was found over the false vocal fold, while the other was found in the right bronchus (in the right lobe). Thirty-five (61.4%) FBs were found in the right main bronchus, 18 (31.6%) were found in the left main bronchus, three (5.3%) were in the trachea, and one (1.7%) FB was found in the larynx located over false vocal cord. Of the removed FBs, 35 (61.4%) were found to be organic in nature and 22 (38.6%) were inorganic. Most organic FBs were corn, orange seeds, vegetables, lemon seeds, watermelon seeds and beans; while inorganic FBs were plastic pieces of toys, metalic parts, nails, buttons, and office pins.

## Discussion

Aspiration and inhalation of FBs are commonly seen events in children that can lead to severe sequelae, especially if not diagnosed (7). The prevalence of trachea-bronchial FBs is greater in children than in adults, particularly below the age of 3 years, as a consequence of behavioral and anatomical characteristics and physiological features (including poor chewing capacity, immature swallowing co-ordination, and high respiratory rates). FB inhalation has a predilection toward male children, which may be due to a higher activity in males (8–10). The course of illness after a FB lodges in the airway passage depends on the FB characteristics and the length of its stay. Undiagnosed, longstanding FBs may lead to complications such as pneumonia, atelectasis, bronchiectasis, and even death (11).

In this study, 60% of patients were under the age of 3 years and 66% were male. This was found to be in accordance with the results of most previous studies (9,12).

The duration elapsed between aspiration of the FB and diagnosis in this study ranged from 2 hours to 5 months. Seventy-five percent of patients presented to us within the first week of FB inhalation, 14.3% presented within the first month, and 10.7% presented later than 1 month. This variability was clearly reflected in the presenting symptoms and signs of the patients. It was noted that patients who presented within the first week with a definite or suspected history of FB inhalation complained of shortness of breath only, except for one patient who complained of stridor and cyanosis due to a FB lodged in the larynx. Symptoms and signs such as cough, fever, and chest rhonchi were seen in patients who were presented later. These finding are consistent with a study reported by Sersar et al. (13), although the latter study was conducted in both children and adults.

Tokar et al. (14) reported that diagnosis is further impeded by the fact that radiolucent FBs (comprising food) represent a large proportion of the pediatric inhaled objects, and are not seen on X-ray in 20 to 35% of cases. This is consistent to a great extent with the present data, although the percentage of patients who showed a normal X-ray was even higher in our study (60.7%).

For proper management of a potentially obstructed air passage, good communication and cooperation between the surgeon and anesthesiologist are mandatory. A detailed operative and anesthetic plan should be discussed before induction. The choice of induction is dominated by the concern of changing a proximal partial obstruction into a complete obstruction. The conversion from spontaneous negative pressure breathing to positive pressure ventilation theoretically has a risk of dislodging an unstable proximal FB to incur complete obstruction.

An interesting finding was noted during bronchoscopy in this study, as 57 FBs were detected in 56 patients, due to two FBs in the form of orange seeds being

extracted from one patient. One of the orange seeds was found over the false vocal fold, while the other was found in the right bronchus. The present data are consistent with some previous studies (14,15) in that the FB usually enters the right bronchus (61.4% in our study), possibly because of its anatomical location (15). In contrast, other investigators found the FB more frequently to pass to the left bronchus, perhaps as a result of the tendency of children to inhale while lying down and holding the FB in their right hand (16). This position also causes a slight straightening of the angle between the trachea and the left bronchus.

The retrieved FBs in this study were mostly organic in nature (61.4%), rather than inorganic (38.6%). These proportions were found to be in accordance with most previous studies (13,17–19). Organic FBs are known to cause irritation of the bronchial mucosa, resulting in a severe local inflammatory reaction and edema. Children who inhale organic FBs usually show manifestation of more intense respiratory distress, demanding urgent management (20). Local inflammation, edema, cellular infiltration, ulceration, and granulation tissue formation may contribute to the obstruction of the airway, making bronchoscopic identification and extraction of the FB difficult. In addition, there is an increased possibility of bleeding in airway on manipulation, allowing the FB to be obscured (21).

Laryngo-tracheo-bronchial FB aspiration must be considered as a matter of urgency in these cases, and must be taken seriously whether there is a definite history or suspected FB aspiration. Early diagnosis and management decrease the incidence of complications and make removal of the aspirated FB easier before development of complications, especially in the case of organic FBs. Laryngo-tracheo-bronchial FB aspiration is better avoided than treated. Preventive measures should be directed at public awareness to decrease the incidence and limit the morbidity of this domestic accident. In addition, medical practitioners must be aware of early referral of patients with suspected FB inhalation.

## Conclusions

FB aspiration is a life-threatening clinical situation, with children <3 years of age being most commonly affected. FB aspiration must be considered a matter of emergency, especially for organic FBs. This study aims to increase the awareness of laryngo-tracheo-bronchial FBs, as early diagnosis and management decrease the incidence of complication and facilitate removal of aspirated FB.
